# A Concealed Typical AVNRT: U Wave Contribution in Simulating Long RP Supraventricular Tachycardia

**DOI:** 10.1002/ccr3.72896

**Published:** 2026-08-04

**Authors:** Gabriela‐Elena Marascu, Alexandru Deaconu, Viviana Alexandra Gondos, Radu‐Gabriel Vatasescu

**Affiliations:** ^1^ Faculty of Medicine Carol Davila University of Medicine and Pharmacy Bucharest Romania; ^2^ Department of Cardiology Clinical Emergency Hospital of Bucharest Bucharest Romania; ^3^ Department of Medical Electronics and Informatics Polytechnic University of Bucharest Bucharest Romania

**Keywords:** case report, electrophysiological study, supraventricular tachycardia, typical atrioventricular nodal reentrant tachycardia, U wave

## Abstract

Accurate identification of P waves on electrocardiogram (ECG) is essential in supraventricular tachycardias because U waves may resemble retrograde P waves, producing a pseudo–long RP interval and hiding typical atrioventricular nodal reentrant tachycardia (AVNRT). A 40‐year‐old woman presented with regular narrow‐complex tachycardia with a positive deflection immediately following the T wave, creating the impression of a long RP tachycardia. The electrophysiological study revealed a short VA interval with an H‐A‐V activation sequence, characteristic of typical slow‐fast AVNRT. This case emphasizes the diagnostic challenge posed by U waves at higher heart rates, which can obscure the true arrhythmia mechanism.

## Key Clinical Message

A typical slow‐fast atrioventricular nodal reentrant tachycardia may be misidentified as a long RP interval tachycardia when U waves resemble P waves. A comprehensive electrocardiogram analysis during both tachycardia and sinus rhythm, along with the electrophysiological study, improves the diagnostic accuracy of supraventricular tachycardias.

## 1 Introduction

Careful analysis and identification of the P wave on the standard 12‐lead electrocardiogram (ECG) is essential in the differential diagnosis of supraventricular tachycardias (SVTs). Less frequently, U waves may mimic retrograde P waves and create the false impression of a long RP interval, thereby concealing typical atrioventricular nodal reentrant tachycardia (AVNRT) [1, 2].

In cases of ambiguous surface ECG findings, intracardiac electrograms obtained during the electrophysiological study (EPS) may help establish the correct diagnosis. During EPS, a VA interval of ≤ 70 ms is characteristic of typical AVNRT, but it may also be observed in focal atrial tachycardia and, less frequently, in orthodromic atrioventricular reentrant tachycardia (AVRT) [1, 3].

We report a rare case of typical slow–fast AVNRT masquerading as a pseudo–long RP SVT on the surface ECG.

## 2 Case History/Examination

**FIGURE 1:**
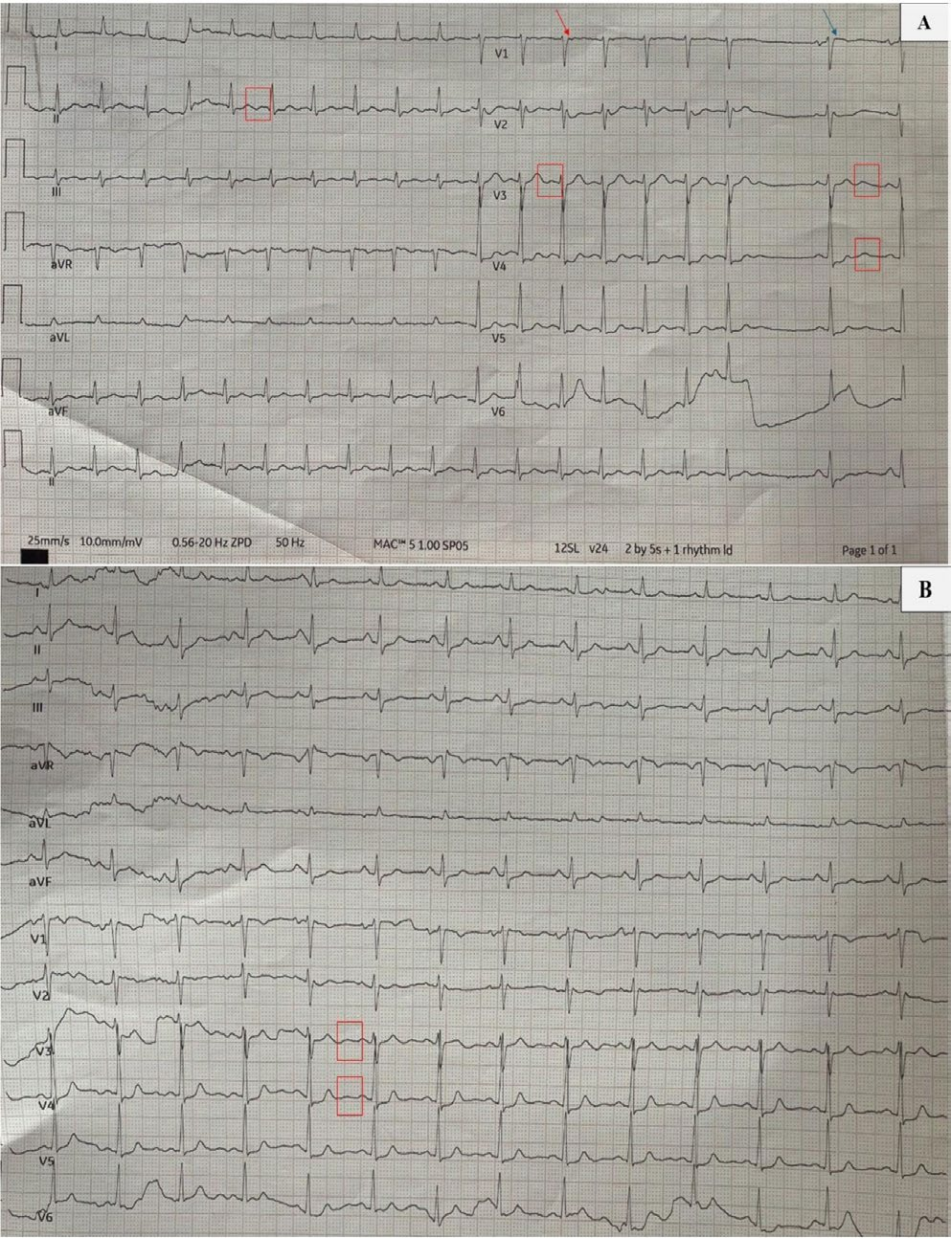
The 12‐lead electrocardiogram (ECG): During tachycardia, the ECG shows a regular narrow QRS tachycardia at 130 bpm with an apparently long RP interval; the red arrow indicates a positive P wave after the QRS complex, which disappears in sinus rhythm (indicated by blue arrow) (A); The red box highlights the U wave immediately following the T wave in tachycardia (A) and sinus rhythm (B).

A 40‐year‐old woman presented with a regular narrow‐complex tachycardia at 130 bpm, with a positive deflection immediately following the T wave during both tachycardia and sinus rhythm, corresponding to a U wave. This mimicked a pseudo‐P wave, creating the impression of a long RP tachycardia, which suggested atypical AVNRT, atrial tachycardia, or permanent junctional reciprocating tachycardia (PJRT) (Figure 1A,B). The patient had a 6‐year history of paroxysmal palpitations characterized by a rapid, regular rhythm with abrupt onset and termination. The episodes had progressively increased in frequency and severity, and she was now experiencing them daily. At presentation, she reported palpitations without associated syncope or chest pain. Physical examination during tachycardia revealed a regular heart rate of approximately 130 bpm, and the patient was haemodynamically stable. The tachycardia terminated spontaneously without the need for pharmacological or electrical cardioversion.

### 2.1 Differential Diagnosis, Investigations, and Management

**FIGURE 2:**
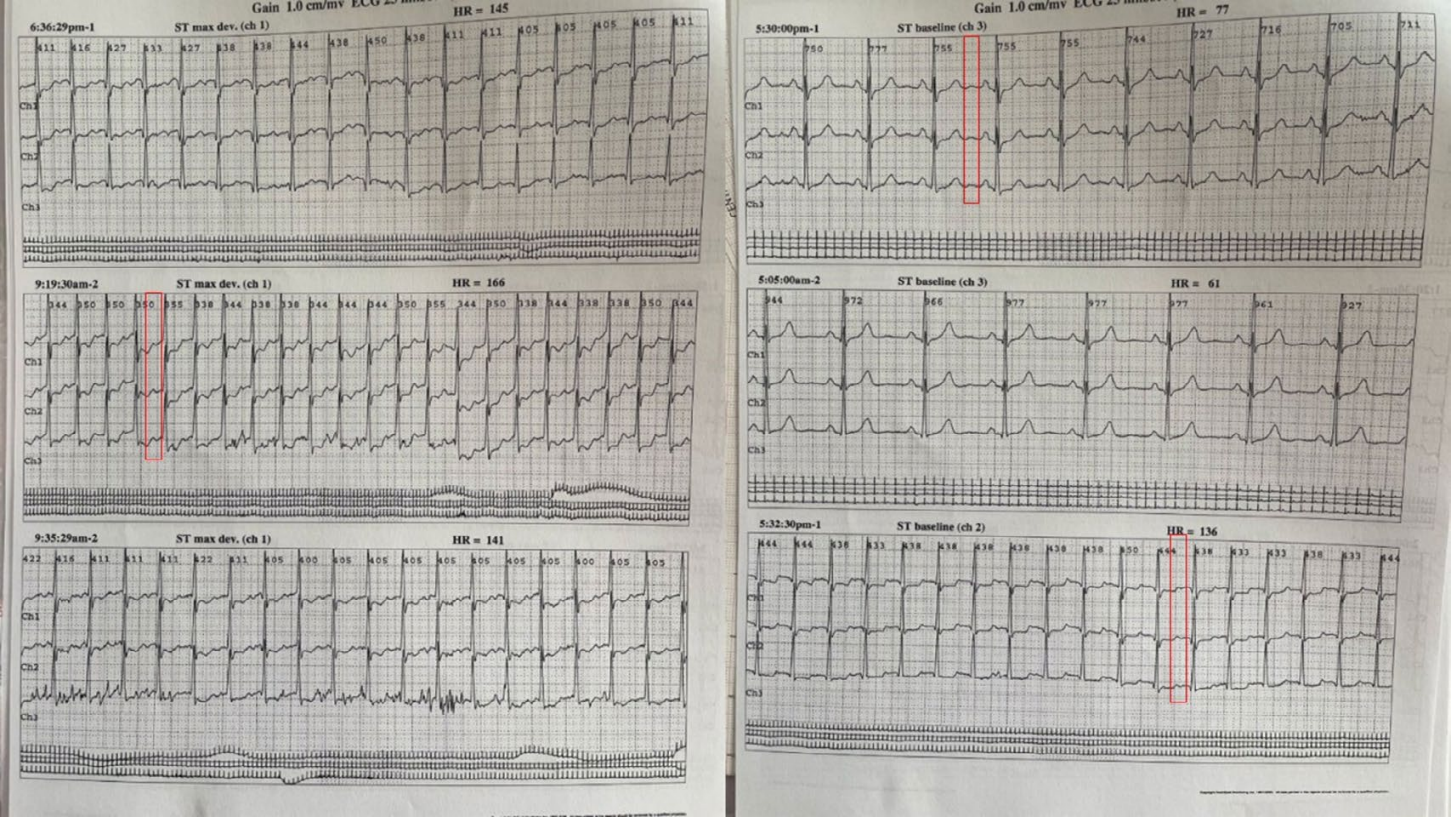
The 24 h‐Holter ECG monitoring; The red box indicates the additional positive deflection after the T wave, resembling a U wave that mimics a long RP interval.

The 24‐h Holter ECG monitoring revealed multiple short episodes of regular narrow‐complex tachycardia at approximately 150 bpm (Figure 2). Transthoracic echocardiography showed no structural heart disease. Laboratory investigations, including complete blood count, renal function, thyroid function tests, and serum electrolyte levels (including potassium and magnesium), were within normal limits. No antiarrhythmic drug therapy was initiated prior to the electrophysiological study (EPS).

**FIGURE 3:**
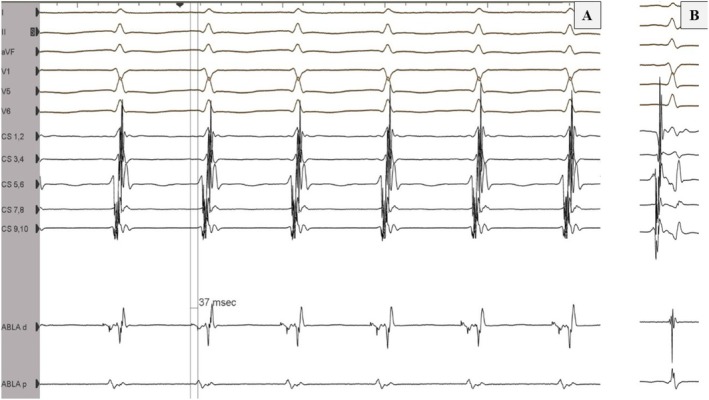
The electrophysiological study performed during tachycardia (A) and in sinus rhythm (B) demonstrated a short VA interval (37 ms) and an H–A–V activation sequence during tachycardia (A), findings consistent with typical slow–fast atrioventricular nodal reentry.

**FIGURE 4:**
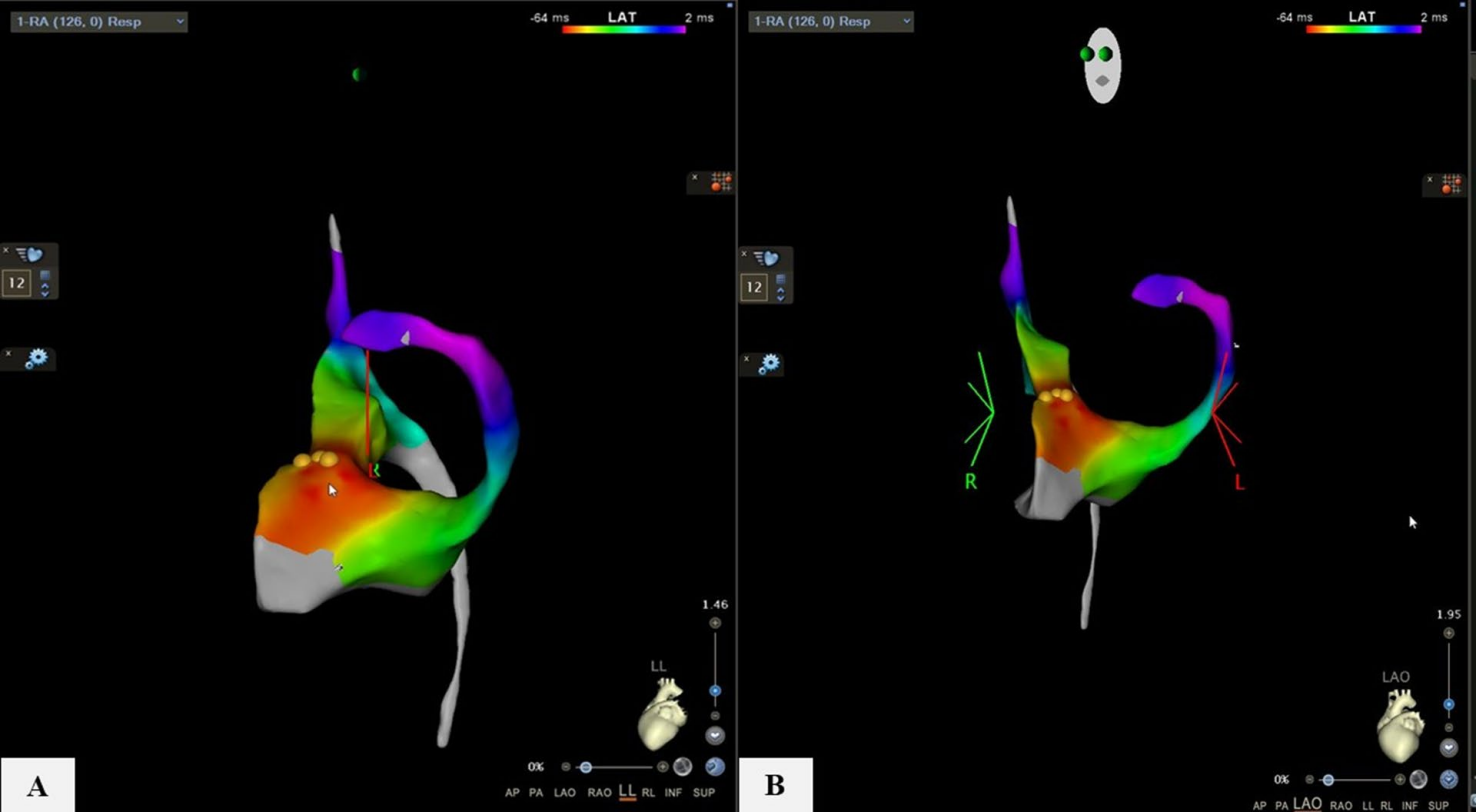
The right atrial and coronary sinus 3D activation of the tachycardia map in the left lateral (A) and left anterior oblique incidence (B) shows first activation of the right atrium in the superior septal aspect, in the vicinity of the His Bundle (yellow dots), which is consistent with the fast pathway exit point.

The EPS performed during tachycardia demonstrated a short VA interval (37 ms) with an H–A–V activation sequence, findings consistent with typical slow–fast AVNRT (Figure 3). The activation sequence during the tachycardia confirms first activation of the right atrium in the superior septal aspect, in the vicinity of the His bundle, which is consistent with the fast pathway exit point (Figure 4).

The patient underwent successful radiofrequency catheter ablation of the slow pathway. The procedure resulted in termination of the arrhythmia, and the patient remained symptom‐free thereafter. No further episodes of supraventricular tachycardia were reported during follow‐up.

## 3 Discussion

Careful analysis and identification of the P wave on the standard 12‐lead ECG is essential in the differential diagnosis of supraventricular tachycardias (SVTs). These arrhythmias are categorized based on the relationship between the P wave and the QRS complex. In short‐RP SVT, the RP interval is less than half of the RR cycle length. In contrast, long‐RP SVT is characterized by an RP interval that is equal to or longer than the PR interval [1]. Less frequently, U waves may mimic retrograde P waves and create the false impression of a long RP interval, thereby concealing typical AVNRT [1, 2]. In cases of ambiguous surface ECG findings, intracardiac electrograms obtained during the EPS may help establish the correct diagnosis. During EPS, a VA interval of ≤ 70 ms is characteristic of typical AVNRT, but it may also be observed in focal atrial tachycardia and, less frequently, in orthodromic atrioventricular reentrant tachycardia (AVRT) [1, 3].

Our case highlights the diagnostic challenge of SVTs. The surface ECG during tachycardia suggested the presence of a long RP SVT, either atypical AVNRT, atrial tachycardia, or even PJRT (which was further supported by the clinical presentation of multiple daily episodes). A detailed analysis of the ECG, which included tracings obtained during sinus rhythm, revealed a positive U wave that followed the T wave. This created the appearance of a retrograde P wave, leading to a misleadingly long RP interval. As a result, the actual typical slow–fast AVNRT was obscured and later confirmed during EPS. Bogossian et al. reported a similar phenomenon, where a U wave appearing during tachycardia simulated a pseudo‐P wave [2]. In their case, U waves were apparent exclusively during the tachycardia and not seen in sinus rhythm. They proposed that alterations in repolarization during tachycardia, driven by mechanoelectrical feedback, are responsible for producing the U waves. By comparison, in our case, the U waves are visible both in sinus rhythm and during tachycardia.

The electrophysiological origin of the U wave remains uncertain and widely debated. Ongoing discussion centers on whether the normal U wave arises from late repolarization of M‐cells, electromechanical stretch of the ventricle, or from small variations in the terminal phases (3 or 4) of the action potential [4]. The U waves are common at low heart rates (< 65 bpm), less frequent at 65–95 bpm, and often obscured at higher rates by broad P waves [4, 5].

Assessment of the baseline ECG alongside the tachycardia tracing may yield important clues to the underlying mechanism of arrhythmias. In our case, the identification of U waves in sinus rhythm facilitated the recognition of pseudo‐P waves as being due to the contribution of U waves.

## 4 Conclusions

A typical slow–fast AVNRT can be misdiagnosed as a long RP tachycardia when U waves mimic the P waves. Accurate diagnosis requires careful ECG analysis during both tachycardia and sinus rhythm, along with EPS.

## Author Contributions

**Gabriela‐Elena Marascu:** conceptualization, data curation, methodology, resources, writing – original draft. **Alexandru Deaconu:** conceptualization, investigation, visualization, writing – review and editing. **Viviana Alexandra Gondos:** data curation, investigation, software. **Radu‐Gabriel Vatasescu:** conceptualization, methodology, supervision, validation, visualization.

## Funding

The authors have nothing to report.

## Disclosure

The authors have nothing to report.

## Consent

Written informed consent was obtained from the patient for publication of this case report.

## Conflicts of Interest

The authors declare no conflicts of interest.

## Data Availability

The authors have nothing to report.
